# The Influence of Irrelevant Visual Distractors on Eye Movement Control in Chinese Children with Autism Spectrum Disorder: Evidence from the Remote Distractor Paradigm

**DOI:** 10.1007/s10803-019-04271-y

**Published:** 2019-10-31

**Authors:** Li Zhang, Guoli Yan, Li Zhou, Zebo Lan, Valerie Benson

**Affiliations:** 1 Academy of Psychology and Behaviour, Tianjin Normal University, Tianjin, People’s Republic of China; 2 Center of Collaborative Innovation for Assessment and Promotion of Mental Health, Tianjin, People’s Republic of China; 3 School of Psychology, University of Central Lancashire, Preston, PR1 2HE UK

**Keywords:** ASD, Visual distractor, Eye movement control, Remote distractor effect

## Abstract

The current study examined eye movement control in autistic (ASD) children. Simple targets were presented in isolation, or with central, parafoveal, or peripheral distractors synchronously. Sixteen children with ASD (47–81 months) and nineteen age and IQ matched typically developing children were instructed to look to the target as accurately and quickly as possible. Both groups showed high proportions (40%) of saccadic errors towards parafoveal and peripheral distractors. For correctly executed eye movements to the targets, centrally presented distractors produced the longest latencies (time taken to initiate eye movements), followed by parafoveal and peripheral distractor conditions. Central distractors had a greater effect in the ASD group, indicating evidence for potential atypical voluntary attentional control in ASD children.

**Electronic supplementary material:**

The online version of this article (10.1007/s10803-019-04271-y) contains supplementary material, which is available to authorized users.

Autism spectrum disorder (ASD) is a lifelong neurodevelopmental condition characterized by social and communicative impairments, and stereotypical interests or behaviours (American Psychiatric Association 2013). Previous studies have shown that individuals with ASD have significant impairments in inhibitory control (IC), for example, individuals with ASD have been reported to perform more poorly when required to ignore task-irrelevant stimuli or to make a prepotent response (evidenced by longer reaction times or increased errors), compared to a typical population (Geurts et al. 2014). These differences could have a negative influence on the development of social and cognitive functioning in ASD since typical development in these domains requires appropriate temporal responses to detect, process and react to important stimuli in the environment. There is also evidence that IC differences, such as increased errors in antisaccade tasks, and attentional capture by irrelevant distractors, in ASD, are associated with symptom severity (Mosconi et al. 2009; Keehn et al. 2016).

The term IC includes a wide range of subcomponents (Christ et al. 2011; Adams and Jarrold 2012). The ability to suppress irrelevant distractors (a subcomponent of IC) seems to be selectively impaired in ASD (Christ et al. 2007, 2011; Adams and Jarrold 2012; Parsons and Carlew 2016). Difficulties in suppressing interference from irrelevant visual distractors in ASD have been consistently demonstrated in a range of tasks (Dawson and Lewy 1989; Burack 1994; Henderson et al. 2006; Dichter and Belger 2007; Remington et al. 2009; Keehn et al. 2010; Fan et al. 2012; Kelly et al. 2013; Remington et al. 2012a; Sanderson and Allen 2013). Specifically, participants with ASD show greater distractor effects in Flanker or in adapted Stroop tasks (in Chevallier et al. 2013) relative to typically developing (TD) participants. In these tasks, the ASD group tends to show longer reaction times or to make more errors than the control groups in incongruent conditions or in distractor trials. It is also suggested that larger distractor effects are particularly apparent in young children compared with older children with ASD (Christ et al. 2011).

In a world comprised of a wealth of competing, often dynamic, visual stimuli, effective target selection coupled with the ability to ignore irrelevant information is essential in the development of higher order cognitive behaviours associated with decision making and social interaction. Inefficiency in the ability to filter visual distractors can lead to inadequate processing of the target and potentially, this could be a contributory factor to the well documented cognitive and behavioral problems associated with ASD (Dawson and Lewy 1989; Burack 1994; Kelly et al. 2013). Previous studies have found close associations between increased failure in target detection and identification tasks (in the presence of visual distractors), and increased ASD symptomatology in the social communication and language domains (Kelly et al. 2013; Keehn et al. 2016).

Considering the potential impact of distracting visual stimuli on information selection and processing in the real world, and the relationship of distractor inhibition deficits and ASD symptomatology, it is important to investigate possible reasons for greater distractor effects in ASD. While, a limited amount of research has attempted to find the cause of this deficit, using a variety of paradigms, the underlying mechanism remains unknown. For example, Burack (1994) views the difficulties as resulting from an inefficient attentional lens. In Burack’s study, autistic participants were found to show inefficiency in focusing attention on centrally presented targets and needed longer to ignore visual distractors in order to make an appropriate response. Based on this evidence, Remington et al. (2009, 2012a, b) investigated whether perceptual load could influence the effect of distractors on performance in ASD. They suggested that greater distractor effects observed in the ASD group could be a consequence of an increased perceptual capacity, and not a failure to filter distractors, as this group were shown to be affected by the presence of distractors in the high level of central perceptual load, even when the TD participants had stopped attending to or processing the distractors in that condition. However, the supportive evidence presented above related exclusively to high functioning adults with ASD, and this finding was not replicated in a later study (Remington et al. 2012a). Additionally, since no eye movements were recorded in these tasks, it is not known whether the ASD group were able to refrain from looking at the distractors. An alternative explanation for the reported enhanced perceptual capacity in ASD could simply reflect difficulties with ignoring the irrelevant distractors, and therefore a failure to focus attention on the central visual task. One way to investigate these issues would be to record and analyse eye movements in ASD.

The relationship between visual attention and eye movements is an essential issue in psychology, and one which has been examined in detail. Shifting attention from one stimulus to another is often accompanied by an overt eye movement, enabling fixation of this stimulus in order to process it in detail (overt orienting, Findlay 2004). Orienting to a stimulus without an overt eye movement is known as covert orienting (Godijn and Theeuwes 2003). When attending to a stimulus, appearance of irrelevant visual stimuli could influence the orienting system at both the overt and covert levels, whereby reflexive eye movements may be directed towards the distractors, rather than to the target, or whereby longer time would be needed to initiate a correct eye movement to the target voluntarily (Benson 2008). The function of eye movements is therefore important in visual attention as the relationship between the two enables us to investigate both voluntary and involuntary attention for target orienting, selection, and the ability to ignore distracting stimuli (Brenner et al. 2007).

ASD has been consistently linked with deficits in eye movement control (Johnson et al. 2016; Baghdadli et al. 2017), including making more saccade errors (Minshew et al. 1999; Goldberg et al. 2002; Luna et al. 2007; Benson et al. 2009; Kelly et al. 2013), or taking longer to disengage from centrally fixated stimuli (Landry and Bryson 2004; Kleberg et al. 2017; Sabatos-Devito et al. 2016) in some intentional saccade tasks. Thus, the larger distractor interference in ASD, observed previously in a variety of paradigms, may derive from attentional impairments which are reflected in reflexive (saccade errors) or voluntary (disengagement speed) eye movement control in these tasks. Longer response time or more errors in RT tasks in ASD, compared to TD performance, may potentially be caused by difficulties in disengaging from distractors when these are fixated, or, alternatively, may be triggered by a failure to inhibit reflexive saccades directed towards the distractors. However, the effects of irrelevant stimuli on the influence of eye movement control in ASD remains unresolved. One study (Kelly et al. 2013), using a search distractor task, specifically investigated this issue and found that group difference effects were absent in the pre-target detection phase, but present in the post-target detection stage, in which the ASD group made more fixations on non-target stimuli after detecting the target relative to the TD group. However, similar eye movement patterns during the first orientation to the target could have resulted from the unique colour of the target making this a simple pop-out search task, and more importantly, it is still unclear as to whether increased distractor effects in ASD, as cited in this introduction, are operating at the reflexive, or the voluntary attentional level.

It is also the case that the distractor studies presented here have tested either adults or older children with ASD. To date no study has investigated eye movement control as a measure of distractibility in younger children. This is important as younger children and even infants with ASD appear to show a different disengagement profile compared to older children or adults. For example, it has been documented that as early as the first year of life individuals with ASD have been shown to display difficulties in attentional disengagement (Elison et al. 2013; Elsabbagh et al. 2013; Bryson et al. 2018), and this observation has been related to the later emergence of ASD symptoms. However, the early disengagement behaviours in infants later diagnosed with ASD seem to reduce as children develop, and older children and adults with ASD have been shown to demonstrate reduced or absent disengagement delay (Johnson et al. 2016). It is possible that this lack of continuity in atypical disengagement across development could be attributed to a transition from underdevelopment of voluntary attentional control in younger individuals with ASD, to an improvement in this area as age increases, although this remains to be empirically tested. In theory, one consequence of an early disengagement impairment could be a failure to detect, and hence respond to available cues in the visual environment, which in turn could lead to a failure to develop ‘typical’ communication skills and hence hamper effective social functioning throughout the lifespan. In support of this idea, the greater distractor effects observed (from the Flanker tasks) in young children (but not older children or adults) with ASD are thought to be underpinned by a developmental delay in voluntary cognitive or attentional control (Christ et al. 2011).

To investigate these issues, the current study utilised a paradigm, known as the remote distractor paradigm (RDP, Walker et al. 1997), to investigate the influence of irrelevant visual information on reflexive orienting (eye movements to the distractor) and voluntary orienting (eye movements to the target) in young children with ASD. Participants are presented either with a single target positioned away from the centre of the display, or they are simultaneously presented with the target and a distractor stimulus. Distractors are presented at either the centre of the display or in the mirror symmetrical location opposite to the target. Participants are instructed to move their eyes to the target as rapidly and as accurately as possible. The findings from the RDP paradigm suggest that saccade latencies (the time taken to initiate an eye movement from the onset of the display) in the distractor trials are longer compared to latencies for the single target trials. This remote distractor effect (RDE) is largest (30–40 ms in Walker et al. 1997) when the distractor is located at the central point of the display, reducing as distractor eccentricity from the centre of the display increases. When the targets and distractors are presented bilaterally, a proportion of exogenous saccade errors (10–30% in Benson 2008), are triggered towards the direction of the distractor, instead of the target. The RDP paradigm has the advantage of enabling investigation of reflexive (exogenous) saccadic orienting (to the distractors—error rates) and voluntary (endogenous) saccadic orienting (to the target—saccade latency) simultaneously in the same trial. The adoption of this paradigm therefore provides an appropriate method to investigate inhibitory and attentional control in ASD.

It is generally accepted that the saccadic orienting system is intact in ASD (Minshew et al. 1999; Luna et al. 2007; Kelly et al. 2013). This is important as any differences in eye movement parameters in the current study between the two groups should reflect attentional or inhibitory differences, rather than any basic eye movement control differences. To ensure that the eye movement system is intact in the young children in our study, basic eye movement control will be established from performance in the main sequence paradigm in Experiment 1, followed by an investigation of distractor influences in ASD as measured from performance in the RDP paradigm in Experiment 2.

In the current paper, in the RDP experiment, we examine the saccade latencies of eye movements initiated towards the target and the proportion of eye movements executed towards the distractors to highlight the aspects of inhibitory attentional control (reflexive or voluntary) that influence performance in young children with ASD. We predict that increased distraction in ASD, at both these levels, will produce more saccade errors to the irrelevant distractors, and also increased saccade latencies to the targets when these are made in the presence of distractors in ASD if both reflexive and voluntary filtering of distractors is impaired in ASD.

## Experiment 1: The Main Sequence Task

### Method

#### Participants

The ASD children (n = 15, age range 40–70 months) were recruited from the rehabilitation institution in Tianjin, China and the TD children (n = 19, 51–73 months) were recruited from one of the local kindergartens.

ASD diagnoses and confirmation: The children with ASD were officially diagnosed with an ASD by at least one experienced clinician, and all met the diagnostic criteria for ASD according to the fifth edition of the Diagnostic and Statistical Manual of Mental Disorders (DSM-V, APA 2013). These diagnoses were confirmed by a hospital staff member, experienced in ASD diagnostic procedures, who examined the records of the children to ensure that they met the DSM-V criteria. Children in the control group were reported to have no history of brain damage or of any neurodevelopmental deficits by their parents. The Chinese version of the Autism Spectrum Quotient: Children version (AQ-child; Auyeung et al. 2008), with a cutoff of 76, was administered to all participants by either parents or teachers. As expected the children with ASD had higher scores on the AQ than the TD children, *t *= 3.43, *p *= .002 (Table 1 shows a summary of the participant AQ scores), and this finding validates the original ASD clinical diagnoses.

**Table 1:** Demographic data (mean ± SD) of the ASD and TD groups in the main sequence task and in the remote distractor task

	The main sequence task				The remote distractor task			
	ASD (n = 15)	TD (n = 19)	*t*-Value	*p*	ASD (n = 16)	TD (n = 19)	*t*-Value	*p*
Age (months)	59.40 (8.57)	62.30 (7.21)	− 1.19	.242	63.69 (9.01)	66.21 (7.01)	− .93	.361
Gender (male/female)	14/1	16/3	*χ*^2^	.613	14/2	16/3	*χ*^2^	1.000
VIQ	111.93 (18.04)	109.89 (9.55)	.42	.674	109.00 (13.13)	108.58 (11.42)	.10	.920
PIQ	108.93 (12.40)	107.53 (9.79)	.37	.714	101.00 (14.40)	105.26 (10.86)	− .99	.326
FSIQ	108.53 (13.98)	112.31 (9.45)	− .94	.354	104.19 (10.04)	110.21 (10.44)	− 1.73	.093
AQ	78.53 (15.37)	63.58 (9.97)	3.43	.002**	79.38 (18.63)	64.37 (9.28)	3.09	.004**

**p < .01

All the children performed a variety of Intelligence tests. Verbal IQ (VIQ), performance IQ (PIQ) and full-scale IQ (FSIQ) were measured by the experimenters using the Chinese version of the Wechsler Preschool and Primary Scale of Intelligence: Fourth Edition (Wechsler 2014). Children with ASD were matched on age, IQ scores and the ratio of gender with the TD children, *ps *> .1. All children from both groups scored in the typical range for all IQ measures (FSIQ scores: 83–122 for ASD group and 91–131 for TD group), and the two groups did not differ significantly from each other in three IQ profiles, *p*s > .05 (Table 1 shows a summary of the participant IQ scores).

The procedures of the current study were approved by the Ethical Committee of Tianjin Normal University. Prior to the study, the parents of all participants were informed of the procedure and informed consent was obtained from the parents of all children participants included in the study.

#### Apparatus

An EyeLink Portable Duo (S.R. Research Ltd, Canada) eye-tracker was used to collect the eye movement data. The sampling rate was 500 Hz. Stimuli were displayed on a 19-inch DELL monitor with a resolution of 1024 × 768 pixels and a refresh rate of 75 Hz. A chin rest was used to maintain head stability throughout testing for all participants.

#### Tasks

The Main Sequence paradigm was utilised to examine basic eye movement control in both participant groups. Participants were asked to look at the single target stimuli which was presented at different eccentricities away from centre of the display. In typical saccadic orienting, there is a linear relationship between saccade peak velocity or saccade duration, and saccade amplitude, in which saccade peak velocity or duration rises as saccade amplitude increases (Bahill et al. 1975; Harris and Wolpert 2006). This positive linear relationship is termed the Main Sequence. The Main Sequence can be used to examine whether eye movements are normal in typical, subclinical or clinical populations (Bahill et al. 1975; Knox 2004).

#### Materials

A simple shape was chosen as the target. This was a yellow circle extending to 1° (31 × 31 pixels). From previous studies, the Main Sequence relationship is normally found when saccade amplitude is less than 15 or 20° (Bahill et al. 1975). In the current study we employed three target eccentricity conditions (4, 8 and 12 degrees) which enabled us to investigate the linear trend of saccade peak velocity or saccade duration against saccade amplitude. To be specific, the centre of the target was located randomly at eight equally separated (compass point) positions around an imaginary circle with a radius of 4°, 8° or 12° eccentricity. Each target position was repeated three times, and there were 72 trials in total.

#### Procedure and Eye Movement Recording

Following verbal instructions the stimuli were presented on paper to participants, so that they could verbally confirm an understanding of the task requirements. A practice session on the eye tracker was also conducted to familiarise participants with this procedure.

In the formal test, a five-point-calibration was performed firstly to measure the position of the eye at different locations on the display screen. The mean error was controlled to be below 0.5° for each child in this process. A small one-point-calibration was used between trials to correct for drifts before the next trial. Each trial began with presentation of a fixation cross (1°) presented at the centre of the black screen for a varying time of 500–900 ms and participants were required to look at the centre of this cross. For each trial sequence the target display was presented for 1200 ms, during which participants were required to look at the centre of the yellow (target) circle as quickly and accurately as possible until it disappeared, followed by a blank screen presented for 500 ms.

#### Eye Movement Measures and Data Analysis

In the main sequence paradigm we recorded and analysed saccade amplitude (angular rotation of the saccade, degrees in visual angle), saccade peak velocity (highest velocity reached during the saccade, °/s), and saccade duration (time to reach the target, ms).

Trials were removed when (1) a blink was made during the trial (*9.08%*), (2) the amplitude of the first saccade to the target was lower than 2° (*4.03%*), (3) the first saccade start position exceeded 1° from the centre of the screen (*3.41%*), (4) an anticipatory saccade (first saccade with latency shorter than 80 ms, Wenban-Smith and Findlay 1991) was made (*1.19%*), (5) the direction of the saccade deviated from target’s direction with more than 22.5° (*1.11%*). In total, 18.82% of the data was excluded, leaving a total of 1976 trials for analyses.

Linear mixed models (LMMs) were performed using the *lme4* package (version 1.1-7) in R (R Development Core 2014) to analyse each of the saccade measures and the main sequence relationships. Here, the LMMs were fitted with random intercept and random slope for the fixed effects over participants only, because the stimuli were the same in each eccentricity. The fixed effects were group and target eccentricity in the analyses of the saccade measures, and group and saccade amplitude in LMM models of the main sequence. Log-transformed data for each saccade measure was analyzed. Absolute values of the t-value (z-value in GLMMs) equal to or greater than 1.96 indicate a significant difference.

### Results

#### Saccade Amplitude

Saccade amplitudes increased as the eccentricity of the target increased. Participants showed the greatest saccade amplitudes in the maximum eccentricity condition, second greatest amplitudes in the middle eccentricity condition and least in the minimum eccentricity condition (|*t|*s > 2). No significant difference between the two groups, nor any group by eccentricity interaction was observed, indicating that both groups showed the same pattern of increased amplitudes to increased target eccentricities.

#### Saccade Peak Velocity and Saccade Duration

Similar results to the saccade amplitude were found for the saccade peak velocity and saccade duration, whereby both groups showed the same patterns of higher peak velocities or longer saccade durations when the target eccentricity increased (|*t|*s > 2). Group differences and the interaction effects of group by eccentricity were not significant (The means and standard deviations of each saccade measure are displayed in Tables 2, 3 shows the results of the fixed effects in LMMs).

**Table 2:** The means and standard deviations of each saccade measure in the main sequence task and the remote distractor task

The main sequence task						
	ASD			TD		
Saccadic measure	4°	8°	12°	4°	8°	12°
Saccade amplitude (°)	3.37 (0.66)	6.70 (1.00)	9.88 (1.53)	3.22 (0.60)	6.49 (1.05)	9.87 (1.32)
Saccade peak velocity (°/s)	176.79 (41.62)	270.48 (51.79)	325.54 (67.33)	178.24 (36.39)	277.15 (55.83)	341.82 (64.34)
Saccade duration (ms)	36 (6)	49 (8)	58 (7)	36 (5)	48 (7)	57 (7)

The remote distractor task								
	ASD				TD			
	C	NR	FAR	ST	C	NR	FAR	ST
Saccade latency (ms)	361 (124)	270 (96)	269 (95)	198 (68)	303 (129)	272 (113)	267 (99)	181 (60)
Error rate		.45 (.50)	.45 (.50)			.38 (.49)	.45 (.50)	

*C* central distractor, *NR* parafoveal distractor, *FAR* peripheral distractor, *ST* single target

**Table 3:** Fixed effect estimates for the saccadic measures in the main sequence task

Effects	Saccade amplitude			Saccade peak velocity			Saccade duration		
	*b*	*SE*	*t*	*b*	*SE*	*t*	*b*	*SE*	*t*
ASD vs. TD	− 0.02	0.02	− 1.21	0.03	0.04	0.65	− 0.00	0.02	− 0.21
Eccentricity 4° vs. 8°	1.11	0.02	70.50***	0.63	0.02	43.54***	0.47	0.02	27.98***
Eccentricity 4° vs. 12°	0.41	0.01	42.94***	0.20	0.01	20.01***	0.18	0.01	18.77***
Eccentricity 8° vs. 12°	− 0.70	0.01	− 55.71***	− 0.44	0.01	− 36.31***	− 0.29	0.01	− 20.30***
ASD vs. TD × 4° vs. 8°	0.05	0.03	1.49	0.04	0.03	1.39	− 0.01	0.03	− 0.40
ASD vs. TD × 4° vs. 12°	0.04	0.02	1.94	0.03	0.02	1.68	0.02	0.02	0.82
ASD vs. TD × 8° vs. 12°	− 0.01	0.03	− 0.40	− 0.01	0.02	− 0.30	0.03	0.03	1.03
ASD vs. TD				0.04	0.04	1.00	0.01	0.01	0.46
Saccade amplitude (SA)				0.10	0.00	46.50***	0.07	0.00	36.50***
ASD vs. TD × SA				0.00	0.00	0.66	− 0.00	0.00	-0.76

***p < .001

#### Main Sequence

**Fig. 1:**
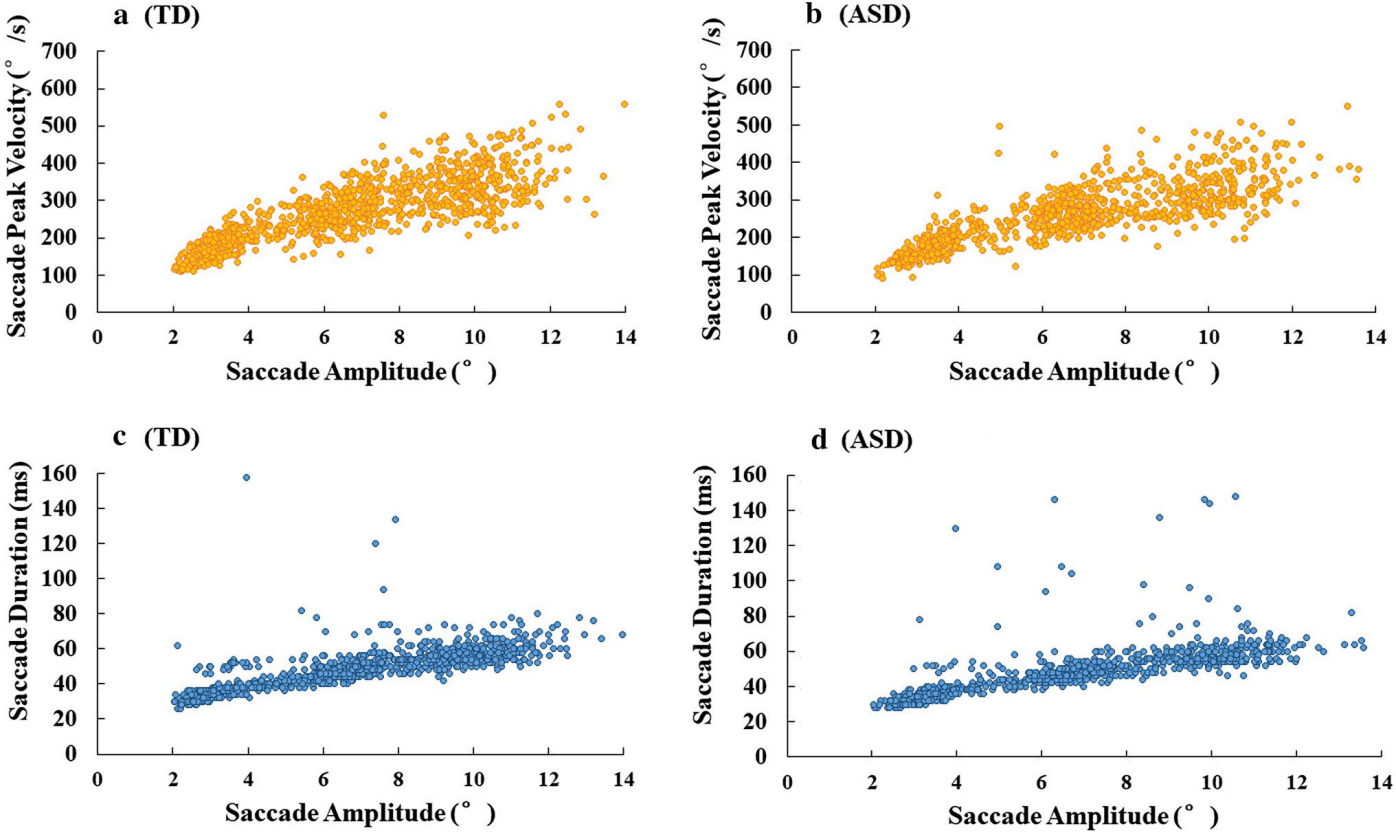
Main sequence relationship between saccade amplitude and saccade peak velocity (**a**, **b**) and between saccade amplitude and saccade duration (**c**, **d**) for both groups

For both groups there were significant effects of saccade amplitude on saccade peak velocity, *b *= 0.10, *SE *= 0.00, *t *= 46.50, and on saccade duration, *b *= 0.07, *SE *= 0.00, *t *= 36.50, which both increased with the increase of saccade amplitude in a stereotypical way. No group difference or interaction effect was found. Overall, the results indicate that saccade peak velocity and saccade duration are closely associated with the saccade amplitude in both groups, increasing linearly as the saccade amplitude increases. Figure 1  shows the main sequence relationships among these saccade measures in both groups.

### Discussion

By adopting the main sequence paradigm we investigated the basic eye movement functioning in young children. Several commonly measured saccadic parameters were compared in both participants groups and the main sequence relationship between saccade peak velocity or duration and saccade amplitude was analysed. Children with ASD showed similar orienting accuracy (saccade amplitude) to the targets in all of the eccentricities compared with the TD children. There was also no difference in the saccade peak velocity and duration between the two groups. More importantly, both ASD and TD children showed the expected main sequence for normal saccadic orienting.

Therefore, in the current study, young TD children and ASD children were shown to have intact basic eye movement control. These findings are consistent with previous reports (Minshew et al. 1999; Luna et al. 2007) and serve to ensure that any differences in eye movement control in future experiments will not result from differences in basic eye movement control in ASD.

In the second experiment the remote distractor paradigm will be adopted to investigate the impact of irrelevant visual distractors on eye movement control in children with and without ASD. By analysing the saccade direction and execution timing in distractor trials, the next experiment permits an investigation of any modulating effects of visual distractors on reflexive and voluntary attentional orienting in typical children, and in children with ASD.

## Experiment 2: The Remote Distractor Task

### Method

#### Participants

Nineteen ASD children (47–81 months) and nineteen TD children (51–74 months) acted as participants. Children with ASD were officially diagnosed with an Autism Spectrum Disorder and all children met the diagnostic criteria for ASD according DSM-V. As with Experiment 1 these diagnoses were confirmed by a hospital staff member, and performance on the AQ scale validated diagnosis, also as in Experiment 1. TD children had no history of brain damage or neurodevelopmental deficits. Prior to the eye movement experiment, the parents of all participants were informed of the procedure, and gave their written consent.

Three ASD participants who failed to complete either the IQ tests or the RDP task were excluded from statistical analyses. The final sample consisted of sixteen ASD children and nineteen TD children. Children with ASD had higher AQ scores (Auyeung et al. 2008) than the TD children, *t *= 3.09, *p *= .004, supporting the original clinical diagnosis of the children with ASD. There was no significant difference in age, IQ scores (WPPSI-IV, 2014) or the ratio of gender between both groups, *ps *> .05 (see Table 1 in detail).

#### Apparatus

The same eye tracker used for the main sequence experiment was used for the RDP experiment. A 24-inch ASUS monitor was used to present the stimuli and this had a resolution of 1024 × 768 pixels with a refresh rate of 144 Hz. A chin rest was used to stabilize head position.

#### Materials

A white square and a white circle were chosen as the target and distractor respectively. The size of each subtended 1°of visual angle (29 × 29 pixels). Targets were presented in isolation or with a central foveal (0° from the centre of the display), parafoveal (4° from the centre of the display), or peripheral distractor (8° from the centre of the display). These three distractor eccentricities were chosen to allow us to investigate the impact of the distractors across the visual field. It is well established that the effect of distractors on saccade latency reduces as distractor eccentricity increases (Walker et al. 1997). The centre of the target was located at an eccentricity of 4° or 8° on the left or right side away from the centre of the screen. Thus, the single target condition and the central distractor condition separately had four target positions. In the parafoveal and peripheral distractor conditions the distractor was displayed at the mirror position opposite to the target. In total there were 12 target positions and each position was repeated 12 times during the experiment. Therefore, each participant completed 144 trials.

#### Procedure and Eye Movement Recording

Participants were given the instructions verbally, followed by a pre-test to validate understanding of the task requirements. Following confirmation of task understanding participants completed a practice session which had the same procedure as the formal experiment, performed on the eye tracker to familiarise participants with the task and procedure.

**Fig. 2:**
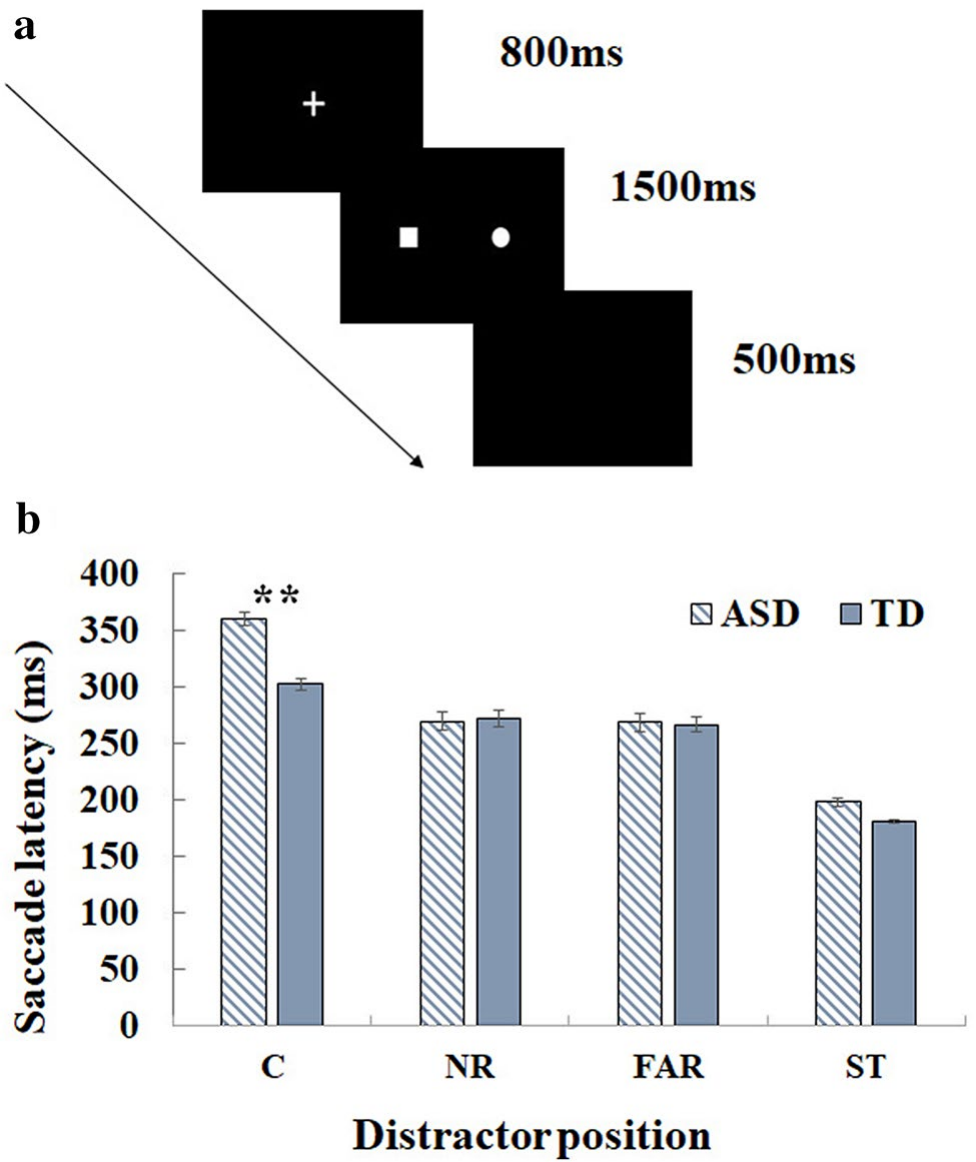
**a** A schematic example of a trial sequence in the Remote Distractor Paradigm (RDP) showing a distractor display where the target and distractor are presented in the parafovea. And **b** the group by distractor position interaction results on saccade latency: *C* central distractor, *NR* parafoveal distractor, *FAR* peripheral distractor, *ST* single target

In the formal testing session a three-point-calibration was performed to measure the position of the eye at different locations on the display screen. Before each trial, a one-point-calibration was presented which participants had to fixate before the trial sequence could begin. This was followed by a white fixation cross (1°) which appeared at the centre of the black screen for 800 ms and participants had to fixate the centre of this cross throughout its duration. Following this, the target display was presented for 1500 ms, during which participants had to look at the centre of the white square as quickly and as accurately as they could, and they were told to ignore anything else that might be presented on the screen at the same time as the target. Finally, in the trial sequence, a blank screen was shown for 500 ms (See Fig. 2 for a schematic of a trial sequence).

#### Eye Movement Measures and Data Filtering

In the remote distractor experiment two measures were analysed (1) The saccade latency for accurate first saccades directed to the target that were greater than 2° (2) the proportion of first eye movements greater than 2°that were executed towards the distractor (errors).

Trials were removed from the final analyses if (1) a blink was made during the trial (*7.23%*), (2) the amplitude of the first saccade to the target was lower than 2° (*6.54%*), (3) the first saccade start position exceeded 1° from the centre of the target display screen (*6.56%*), (4) an anticipatory eye movement was made (*0.93%*), (5) saccade latencies were greater or lower than 3 standard deviations away from the mean value of individual participants (*1.09%*). In total, 3827 trials were included in the final analyses.

LMMs were conducted to analyse the dependent measures. The random intercept and random slope on the fixed effects of distractor position over participant were fitted in LMMs. Saccade latencies were log-transformed to normalize the data and error data were analyzed in the logistic GLMMs.

### Results

Descriptive data with respect to the saccade latencies and error rates for both groups are presented in Table 2. Details of the fixed effects results for both measures can be referred to in the Table 4. Below we report the error data first, followed by the saccade latency data for correct trials.

**Table 4:** Fixed effect estimates for saccadic measures in the remote distractor task

Effects	Saccade latency		
	*b*	*SE*	*t*
ASD vs. TD	− 0.06	0.06	− 1.01
ST vs. FAR	− 0.23	0.03	− 7.11***
ST vs. NR	− 0.23	0.04	− 6.54 ***
ST vs. C	− 0.54	0.02	− 23.60***
NR vs. FAR	− 0.00	0.03	− 0.13
FAR vs. C	− 0.31	0.03	− 11.89***
NR vs. C	− 0.31	0.03	− 9.30***
ASD vs. TD × ST vs. FAR	0.19	0.07	2.84**
ASD vs. TD × ST vs. NR	0.19	0.07	2.68*
ASD vs. TD × ST vs. C	0.10	0.05	2.17*
ASD vs. TD × NR vs. FAR	− 0.00	0.05	− 0.04
ASD vs. TD × FAR vs. C	− 0.09	0.05	− 1.68
ASD vs. TD × NR vs. C	− 0.09	0.07	− 1.33

	Error rate		
ASD vs. TD	− 0.17	0.15	− 1.15
NR vs. FAR	− 0.13	0.12	− 1.12
ASD vs. TD × NR vs. FAR	− 0.28	0.23	− 1.21

*p < .05; ***p < .001

#### Directional Errors

The directional error rate was calculated as the ratio between the error trials and the total valid trials in the parafoveal and peripheral distractor conditions for each participant. There was no significant main effect in either distractor position, *b *= − 0.12, *SE *= 0.11, *z *= − 1.05, or group, *b *= − 0.18, *SE *= 0.15, *z *= − 1.22. The interaction effect did not approach significance, *b *= − 0.29, *SE *= 0.23, *z *= − 1.29.

There was however a high level of directional errors in both groups (ASD: *M *= 45%, TD: *M *= 41%). It was apparent that this saccade task was challenging to these young children aged from 4 to 6 years when the distractors and targets were displayed simultaneously on the screen. Whilst it is possible that the high error rates in children could reflect a misunderstanding of the instructions, we did control for this by asking all the children to tell the experimenter, in their own words, what they were supposed to do in the experiment before the experiment started. One other possibility for the high error rates could reflect a failure to discriminate between the distractor and the target when these were simultaneously presented in the parafovea or periphery. However, a post discrimination test was conducted which confirmed that both groups could tell the shape of the extrafoveal stimulus when they fixated on the central point of the screen (the mean judgement accuracies were above 95% for both groups). This means that the high error rates could not be accounted for by an inability to discriminate between the target and distractor when they were presented together. The high error rates could also result from a less developed eye movement control system in the children. However, the main sequence data indicates that low level eye movement control is intact in the children in the study. Another potential cause of the high error rates could relate to voluntary control. If children have less well developed voluntary control then this might result in random allocation of the eyes to either the target or distractor when these are presented together. This is what we observed in the error rates in the current study. If it is the case that the children have a problem with voluntary control, since we know that the children can discriminate between the target and the distractor, then on error trials, where the eye movement is directed towards the distractor, children should quickly make a second corrective saccade to the target.

In order to investigate this hypothesis, we examined whether corrective saccades to the target were made following an erroneous eye movement towards the distractor in both groups of children. Corrective trials were categorised as those where participants made a first saccade towards the distractor followed by a second saccade to the target which was greater than 2°. The percentage of corrective trials in the parafoveal distractor condition (NR) and the peripheral distractor condition (FAR) were 94.01% and 95.49% for the ASD group, and 98.50% and 96.05% for the TD group, which were significantly greater than chance levels (50%, *ps *= .000). There was no significant difference between the two groups, regardless of the distractor positions, *p*s > .1. The very high proportion of corrective saccades in both groups indicates that children might have a problem with voluntary control over their reflexive eye movement system.

One further analysis was conducted to rule out a lack of task instruction knowledge underpinning the high error rates. This analysis compared the first fixation duration (FFD) on the targets for the first correct response trials (FCR) with the FFD on the distractors for the corrected response trials (CR). The results suggested that the FFD was longer for the FCR trials compared to the FFD for the CR trials in the NR and FAR conditions for both groups (ASD: the FFD difference between FCR and CR was 87 ms in the FAR, *p *= .058, and 190 ms in the NR, *p *= .001; TD: the differences were 173 ms in the FAR, *p *= .000, and 264 ms in the NR, *p *= .000). These results suggest that both groups followed the instructions correctly and the high error rates are likely to reflect random allocation of an eye movement to either the target or distractor when presented together, as a result of a less well developed voluntary attentional control system in children of this age. Previous eye movement control studies with children have also shown support for this (see Luna et al. 2008 for details).

#### Saccade Latency

Basic distractor effects were examined first by comparing the latencies in different distractor eccentricities with those for the single target condition. Participants took longer to initiate an eye movement to the target when it was presented simultaneously with distractors compared to the single target condition, |*t|*s > 2. Additionally, expected RDE effects were observed, whereby saccade latencies in the central distractor condition (*M *= 323 ms, SD = 130 ms) were longer relative to the parafoveal (*M *= 271 ms, SD = 107 ms) and peripheral distractor conditions (*M *= 268 ms, SD = 97 ms) (|*t|*s > 1.96). No significant difference between the ASD and TD groups was found, *b *= − 0.05, *SE *= 0.06, *t *= − 1.01. However, a significant interaction of group by distractor position showed that the ASD group had an increased RDE compared to the TD group. Further analyses revealed that the ASD participants had longer saccade latencies (*M *= 361 ms, SD = 124 ms) than the TD participants (*M *= 303 ms, SD = 129 ms) in the central distractor condition, *b *= − 0.17, *SE *= 0.06, *t *= − 2.78, but performed comparably with the TD group in the other distractor conditions and in the single target condition (see Fig. 2).

In summary, the latency data indicate that there are group differences in the time taken to initiate an eye movement towards the target when the distractors are presented at the central location. The ASD children took longer to look towards the target in the presence of the central distractor. Possible reasons for this will be discussed in the next section.

### General Discussion

The current study utilised the RDP task to investigate the ability to ignore irrelevant distractors in young Chinese children with ASD. Specifically, the remote distractor effects of a simple shape stimulus, presented with the target simultaneously, were examined to reveal whether there were differences between our two participants groups in either the proportion of eye movements directed initially to the distractors (errors) or in the time taken to initiate an eye movement to the targets (latencies).

The error data revealed that the children were randomly allocating attention to either the target or the stimuli when these were presented together. Detailed investigation as to why this might be led to the conclusion that the high (almost 50%) errors likely resulted from an underdeveloped voluntary control system, and this was observed in all the children, from both participant groups. This inference is supported by the findings from the main sequence task which provided support for an intact basic orienting system, and also by the finding that the children quickly executed a corrective eye movement toward the targets following initial eye movements (with short fixations) directed to the distractors.

The results from the main sequence experiment and the error data from the RDP experiment clearly verify that children with ASD have no impairment in their low level orienting system. Importantly, when we analysed the latency data, it was found that the ASD group showed longer saccade latencies relative to the TD group when the target was presented with a central distractor. These results point to a greater disengagement delay from the central distractors in saccadic orienting towards the target in the ASD group, and this finding is consistent with previous reports (Landry and Bryson 2004; Kleberg et al. 2017; Sabatos-Devito et al. 2016). However, the current study makes a novel contribution to the area by providing evidence that the disengagement delay in young children with ASD results from impairments at the voluntary (and not the reflexive) control level. This is revealed in the eye movement data which shows that the time needed to suppress centrally presented irrelevant visual distractors in the RDP, in young children with ASD, is longer compared to TD children. A deficit in disengagement has also been consistently suggested to be one of the earliest markers in infants at-risk for ASD, and, has been shown to predict a later diagnosis prospectively (e.g. Elison et al. 2013; Elsabbagh et al. 2013; Bryson et al. 2018). The consistently observed disengagement delay in infants with high risk for ASD has been shown to impact on the clinical symptoms that manifest in ASD. Recent research has revealed close correlations between the delayed disengagement speed in ASD and atypical responses to sensory stimuli, and, negative emotional patterns of behaviour when confronted with novel or unexpected events in prospective studies (Kleberg et al. 2017; Bryson et al. 2018).

It appears, from the findings from the current and recent studies, that the observation from the previous studies of slowed attentional disengagement in early life, even before a diagnosis of ASD, continues to exist in the young children with ASD. However, the findings from the current study add to our understanding of how IC operates in ASD by showing that impairments in distractibility are exclusively observed for the component of voluntary attentional control in young children with ASD.

Moreover, disengagement delay from the central distractor in ASD during early development cannot be accounted for by deficits in the low-level eye movement system, as we have shown that the ASD participants have intact performance in the main sequence task. The atypical attentional processing observed in the current experiment could reflect atypical development in voluntary attentional control in ASD (Christ et al. 2011). It has been repeatedly suggested that individuals with ASD improve their attentional control with age, performing better at inhibiting irrelevant stimuli or prepotent responses (Luna et al. 2007; Solomon et al. 2008; Geurts et al. 2014; Schmitt et al. 2017) as they develop. Furthermore, research employing older children or adults with ASD has revealed no differences in attentional disengagement processing between ASD and typical control groups (see Johnson et al. 2016 for a review).

The finding that longer time was needed to disengage from the central distractors in ASD, in the current study, indicates atypical voluntary attentional control in that group. This atypicality could help explain the reason for larger distractor effects commonly found in the flanker (or adapted flanker) tasks in ASD. In these tasks, in which the target was presented simultaneously with the bilateral or unilateral distractors, participants with ASD took longer to disengage from fixated distractors. This attentional characteristic could impede orienting to a target quickly and accurately, resulting in longer RT or greater response errors in ASD. Therefore, the delayed attentional disengagement presented in young children with ASD could result in failures to detect or learn the importance of cues presented in the environment, and thus, could be a factor that influences inappropriate responses and decisions when engaged in everyday activities or interactions. Thus, the implications of the findings for the field include an explanation as to how disengagement delays in young children with ASD might lead to impairments in the typical development of communication skills.

Voluntary attentional control is essential for people to detect important information in their environment, in order to be able to react appropriately to the key cues (often non-verbal or implicit) present in everyday communication. It is suggested that neurotypical individuals tend to scan social scenes flexibly and show corresponding eye movement trajectories according to task instructions (Yarbus 1967; Liversedge and Findlay 2000; Benson et al. 2009). However, individuals with ASD do not sample the information presented according to top down requirements (Benson et al. 2009; Birmingham et al. 2011; Riby et al. 2013). Other evidence indicates that although ASD usually attend to visually salient stimuli, they fail to prioritise social information in social scenes (Fletcher-Watson et al. 2009; Amso et al. 2014), while neurotypical individuals will intentionally direct their attention to the social stimuli, regardless of the salience value of the stimuli (Birmingham et al. 2009). Deficits in voluntary attentional control in ASD could result in a failure to detect important communication cues, which can appear and disappear rather quickly, such as facial emotions or eye gaze. The ASD group are found to make less attentional shifts among different talkers in a video clip or a real social context relative to TD group (Klin et al. 2002; Banez et al. 2008). A close relationship between atypical attention patterns to faces and poorer performance in recognizing facial identities or emotion has also been indicated in ASD (Kliemann et al. 2010; Falkmer et al. 2011; Kliemann et al. 2012). It is not yet known how faces might affect performance in the paradigm used in the current experiment, or whether manipulation of emotional expression or eye gaze on such face distractors might modulate performance in children with ASD, in terms of disengagement or other aspects of voluntary attentional control, but these issues should be explored in future work.

One limitation of the current study relates to the diagnostic and confirmation procedures of the ASD individuals. Although diagnoses were confirmed by hospital staff members, and were validated by AQ scores, future studies should aim to adopt established western tests, such as the Autism Diagnostic Observation Schedule (ADOS) or the Autism Diagnostic Interview-Revised (ADI-R) for diagnostic and confirmation procedures in Chinese individuals.

In summary, the findings from the current study, using a very simple eye movement paradigm, indicate that Chinese children with ASD have disengagement difficulties from centrally presented stimuli. Such delayed attentional control in ASD could be a factor that affects abnormalities in social cognition or even social interaction. Failure to quickly disengage and move attention elsewhere might mean that relevant information is ‘missed’ resulting in an inability to respond appropriately or effectively in the everyday communication domain.

## Electronic supplementary material

Below is the link to the electronic supplementary material.

Supplementary material 1 (XLSX 24 kb)

Supplementary material 2 (XLSX 189 kb)

Supplementary material 3 (CSV 60 kb)

Supplementary material 4 (XLSX 40 kb)

Supplementary material 5 (CSV 20 kb)

Supplementary material 6 (CSV 56 kb)
